# Bacteriophages: Combating Antimicrobial Resistance in Food-Borne Bacteria Prevalent in Agriculture

**DOI:** 10.3390/microorganisms10010046

**Published:** 2021-12-27

**Authors:** Arnold Au, Helen Lee, Terry Ye, Uday Dave, Azizur Rahman

**Affiliations:** 1 Centre for Climate Change Research, University of Toronto, ONRamp@UTE, Toronto, ON M5G 1L5, Canada; arnold.au@mail.utoronto.ca (A.A.); hx.lee@mail.utoronto.ca (H.L.); terry.ye@mail.utoronto.ca (T.Y.); uday@climatechangeresearch.ca (U.D.); 2 A.R. Environmental Solutions, ICUBE-University of Toronto, Mississauga, ON L5L 1C6, Canada; 3 Faculty of Arts & Science, University of Toronto, Toronto, ON M5S 3G3, Canada

**Keywords:** agriculture, bacteriophages, food-borne pathogens, antimicrobial resistance, phage therapy

## Abstract

Through recent decades, the subtherapeutic use of antibiotics within agriculture has led to the widespread development of antimicrobial resistance. This problem not only impacts the productivity and sustainability of current agriculture but also has the potential to transfer antimicrobial resistance to human pathogens via the food supply chain. An increasingly popular alternative to antibiotics is bacteriophages to control bacterial diseases. Their unique bactericidal properties make them an ideal alternative to antibiotics, as many countries begin to restrict the usage of antibiotics in agriculture. This review analyses recent evidence from within the past decade on the efficacy of phage therapy on common foodborne pathogens, namely, *Escherica coli*, *Staphylococcus aureus*, *Salmonella* spp., and *Campylobacter jejuni*. This paper highlights the benefits and challenges of phage therapy and reveals the potential for phages to control bacterial populations both in food processing and livestock and the possibility for phages to replace subtherapeutic usage of antibiotics in the agriculture sector.

## 1. Introduction

Antibiotics have been used in animal and crop production as prophylaxis to minimize the risk of disease, increase weight gain livestock, and enable confined livestock production. Due to the improper use of antibiotics, there has been an increase in antimicrobial resistance (AMR) in food products and animals, resulting in a spike in foodborne bacteria. Consequently, more potent antibiotics need to be developed, but their subsequent misuse only results in a positive feedback loop cultivating stronger AMR. This impacts the control of disease in the food production industry, mainly affecting the health and productivity of livestock. Studies have found the presence of AMR in animal products, suggesting the possibility of gene transfer of antimicrobial resistance genes (ARGs) from agriculture to human pathogens and contributing to the development of AMR in clinical diseases [1,2]. Furthermore, traditional agricultural practices, such as manure fertilization, have led to the transfer of large amounts of antibiotics into the environment (resulting in ideal conditions for bacteria to cultivate additional AMR). Manure also provides an environment for horizontal transfer of ARGs to other pathogenic species not only in the surrounding environment but also into downstream freshwater systems [3]. Therefore, ARGs for diaminopyrimidine, quinolone, and tetracycline have been transferred from wastewater into nearby irrigated soils, groundwater, and sewage treatment plants, which have the potential to transfer to human communities [3]. Thus, there has been increased research on alternatives to antibiotics while maintaining health and productivity.

**Figure 1:**
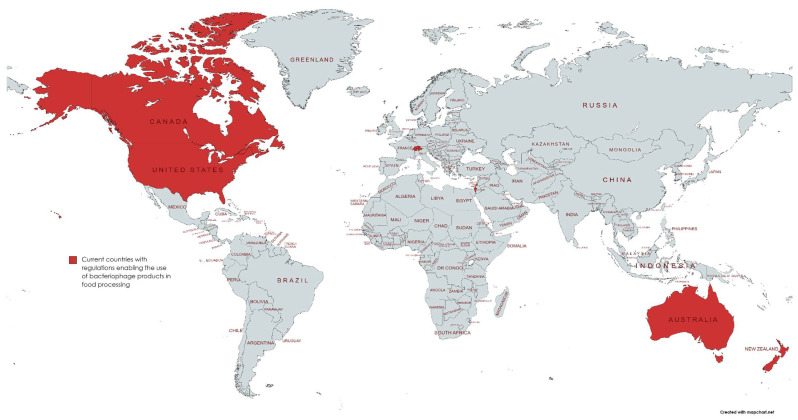
Map of current countries with regulations enabling the usage of phage products in food processing and food products. Countries include Canada, the USA, Switzerland, Israel, Australia, and New Zealand.

While there have been various approaches to reduce usage through diet control, feeding of probiotics and the use of vaccines, a particular approach that is gaining interest is the use of bacteriophages to target bacteria. Bacteriophages, more commonly referred to as phages, are a class of viruses discovered for their bactericidal effects even before the discovery of penicillin and other antibiotics. However, due to the ease of use and importance of antibiotics in World War II, research on bacteriophages stagnated until the recent postantibiotic era and the increasing severity of AMR. As viruses that target only a specific family of bacteria, phages effectively kill colonies of infection-causing bacteria while ignoring other beneficial microorganisms and cells. Regulations for phage therapy for human application are strict due to limited clinical trials on the safety of humans; however, several countries have passed regulations allowing for the use of phage therapy in food processing and agriculture (Figure 1). While there are currently no regulations allowing for phage product use within the European Union, phage products are allowed on food exports to countries where it is authorized. Health Canada has issued Letters of No Objection for bacteriophage mixtures against *Escherichia coli* (*E. coli*), *Salmonella enterica* (*Salmonella*) and *Listeria monocytogenes* (*Listeria*) as processing aids for the removal of pathogenic *Salmonella* from meat products post-slaughter and processing [4]. EcoShieldTM and PhageGuard ETM, among other bacteriophage mixtures against *E. coli*, *Salmonella* and *Listeria,* are approved in the United States by the FDA for use in packaged meat and food products. As more investigations and assessments on the risks of phage therapies are conducted, it will not only improve the situation of AMR in the food supply chain but also provide evidence for the potential safety of use in treating bacterial infections in humans.

This paper will compare the bactericidal effects of phage therapy to antibiotics by focusing on several bacteriophage strains that target common epidemiological pathogenic bacteria in agriculture that have already or are at risk of developing AMR. Through analysis of the evolution of AMR in agriculture, the bactericidal effects of bacteriophages, the possibility of phage resistance, and current challenges to phage therapy, this paper will compare phage therapy to agricultural antibiotics and the potential to replace or work in tandem with antibiotics to protect the health and productivity of agriculture.

## 2. Antimicrobial Resistance in Agriculture

The crisis of antibiotic resistance has been a growing concern since the discovery of penicillin and is being addressed by the continued development of new antibiotics. The evolution of AMR is not exclusive to human pathogens and has been observed in animals and food products from agriculture. Especially within the last decade, an increased number of *Salmonella* and *E. coli* isolates from humans, food products, and animals were found to be resistant to at least 6 or more classes of commonly used antimicrobials. This is most evident with the discovery of a new highly drug-resistant *Salmonella* strain found in poultry for the first time in 2018 [5,6]. The sole reliance on antibiotics has proven to be unsustainable, most evident by not only the increase in resistance but also the declining rate of development of new antibiotics. Only eight have been approved since 2017, with limited clinical benefits over current antibiotics. Additionally, only six are in development that fulfills at least one of the innovation criteria set by the WHO [7].

The literature from the past decade shows that the development of AMR is increasing in pathogenic food-borne bacteria from different agricultural sources, with some strains becoming resistant to several classes of antimicrobials. This is dangerous, as these resistant bacteria are already infectious to humans and will bring new ARGs to the gene pool of food-borne bacteria.

*E. coli* is a gram-negative bacterium commonly found in the digestive tracts of animals, particularly in agricultural livestock, such as cattle, sheep, swine, and poultry, as well as contaminated meat products. *E. coli* O157:H7 is the most common foodborne Shiga toxin-producing *E. coli* strain that causes gastrointestinal diseases and stereotypical food poisoning symptoms, such as diarrhea and vomiting. *E. coli* is growing in prevalence in agriculture and has been reported in dairy farms infecting the microbiomes of cattle [8]. This is additionally problematic, as these pathogens have been known to be shed in their feces, contaminating the environment and spreading to others. Multiple studies of AMR in *E. coli* have reported moderate to severe resistance to several antibiotics used to treat cases of *E. coli* infection, including streptomycin (~30% to ~92%) and ampicillin (~16% to ~63%), and high rates of multidrug resistance to several classes of antibiotics (~20% to ~92%), with over 50% of cattle-infecting strains possessing MDR to seven antimicrobial classes, all of which are also used for treating human clinical cases of *E. coli* infection [8,9,10,11,12].

The *Staphylococcus* genus is a common bacterium impacting human and animal health. *Staphylococcus aureus* is one of the main culprits causing mastitis and inflammation of breast tissue. The spreading of *S. aureus* between cattle by contaminated milk has greatly impacted milk production and yield in the dairy industry. Methicillin-resistant *Staphylococcus aureus*, MRSA, has increased in recent years and is a major cause for concern, as MRSA is resistant to a majority of common antibiotics used in treating both humans and animals. Several agricultural studies have reported increased resistance in *S. aureus* to common antimicrobials, penicillin (48% to 90%), and moderate resistance to tetracyclines (39% to 56.1%), which are antibiotics commonly used in dairy and swine agriculture [12,13,14,15,16]. As a consequence, these AMR profiles have leaked into local environments and frequently transferred to workers in the agricultural sector, such as farmers and veterinarians. This resulted in a higher prevalence of tetracycline and clarithromycin resistance in *S. aureus* infecting swine farmers [13]. This demonstrates the disastrous consequence of AMR development and spread in the environment.

*Salmonella* spp. can infect cattle, poultry, and swine and thus is prevalent as a foodborne pathogen in uncooked meat products, causing symptoms of gastroenteritis and typhoid fever in humans. Swine and poultry are typical hosts and reservoirs for several strains of antibiotic-resistant *Salmonella*, including *enterica* and its serovar Typhimurium. *Salmonella* is commonly found in raw pork, poultry meat and eggs but has also been found to be present in produce, likely due to the application of contaminated feces, such as manure, contaminating local soil environments. *Salmonella* can enter human populations through the consumption of improperly cooked contaminated food products, as well as contaminated handling within food processing plants [17]. Specific Salmonella serovars, such as Heidelberg and Typhimurium, are found in both poultry and humans. These serovars are closely related genetically and are capable of sharing plasmids containing ARGs. This supports the notion that multidrug resistance from poultry-sourced Salmonella is capable of transferring human-infecting Salmonella, affecting the treatment of Salmonella infections in humans [17,18]. Reports of increasing AMR in *Salmonella* are appearing in both agriculture and humans, with high resistance, especially to amoxicillin (20% to 100%) and streptomycin (16.8% to 67.5%), which in addition to treating *Salmonella* infections is also used to treat a variety of other illnesses in humans [8,17,18,19,20,21].

*Campylobacter jejuni (C. jejuni)* is a common foodborne pathogen found in developed and developing countries and is prevalent in undercooked poultry meat products, raw dairy products, raw vegetables, and contaminated water. *C. jejuni* has been found to be capable of surviving in local environments, employing a variety of mechanisms, such as superoxide dismutase enzymes and biofilm formation, to persist in livestock wastewater, which transfers *C. jejuni* populations into local soils and water systems [22]. This increases the risk of transmission between livestock and pollutes local environments for potential transfer to other animal species as well as humans. This raises additional concerns, as *C. jejuni* has developed resistance to many of the common antibiotics used in clinical treatments, such as ciprofloxacin (6.9% to 53.9%) and tetracyclines (47% to 60%) [23,24,25,26] and has been observed to persist in areas surrounding farms, despite prophylactic antibiotic restrictions, suggesting transfer of ARG in local *C. jejuni* populations [26,27].

The lack of veterinary surveillance and unregulated use of subtherapeutic doses of antibiotics are widely believed to be the main contributors to AMR development within livestock populations. Studies have demonstrated high levels of AMR in poultry-isolated and swine-isolated *Salmonella* within livestock and areas surrounding farms that use antibiotics for subtherapeutic use. It was suggested that the high prevalence of AMR was due to the ability of antibiotics and resistant pathogens to persist within feces, cultivating AMR for months outside animal hosts [28,29]. The evidence for the rise in AMR of several bacterial strains prevalent in agriculture highlights the crisis of unregulated antibiotic usage in agriculture in this sector. In addition to affecting food-borne illnesses, the agricultural use of the same antibiotics used to treat clinical cases has been suggested to result in a zoonotic transfer of AMR to human-infecting pathogens [12,24,27], with *S. aureus* isolates from pig farmers possessing more resistance to tetracyclines than nonrelated personnel [13]. Despite the discontinuation of antibiotics sub-therapeutically, the consequences can persist in human populations and local soil environments for extended periods.

A common agricultural practice is applying animal feces as fertilizers. The prevalence of AMR persistence in feces enables AMR to evolve and provide ARGs in bacteria the opportunity to share their ARGs with soil microbiomes through horizontal gene transfer and DNA plasmids [28,30]. Although practices, such as manure composting rely on high temperatures (~55) to remove pathogens, it enables ARGs to persist and spread. Manure composting was found to significantly reduce tetracycline-resistant *E. coli* populations, but tetracycline-resistance genes were still detected at low concentrations and found to persist for at least 100 days [31]. Spores of multidrug-resistant *Clostridium difficile* were found to be resistant to manure composting conditions of 60 °C for 5 days, requiring higher temperatures and/or longer heat treatment periods than current guideline recommendations [32]. Although it is possible to reduce the number of ARGs, complete eradication is challenging, and there is still a risk of gene transfer to other bacterial species in the soil [31,32]. Tetracycline resistance genes were discovered in local soil microbes in areas that had manure fertilizer applied, as well as in alternative species of soil microbes found up to 100 m away from the farm [28]. Additionally, a study performed on one of the swine farms discovered the persistence of ARGs in the local environment 6 months after the last application of antibiotics. Further in vitro experiments suggest that these ARGs could potentially remain for up to 18 months after the discontinued use of antibiotics [28]. Even with proper disinfection of meat products, AMR can be cultivated in agricultural soils and enter the food supply chain through vegetable microbiomes. This was demonstrated through the application of poultry and cattle manure in a lettuce field. The lettuce was grown using soil that contained an increased presence of ARGs and plasmids in lettuce tissues, which matched the soil microbiome [33]. Thus, the pollution of livestock waste containing excess antibiotics has led to a deeper spread of AMR in the environment that is proving difficult to remove.

Countries, such as the Netherlands are beginning to regulate the use of subtherapeutic antibiotics in livestock. In 2014, the Netherlands regulated the use of fluoroquinolone antibiotics to require veterinary approval and only after all other options were exhausted. However, despite these strict regulations and a significant reduction in resistance proportions, AMR continues to persist after more than two years after discontinued usage, and proportions are still comparable to the resistance proportions at the beginning of the century [34]. While the regulation effectively reduced *Salmonella* and *E. coli* in livestock, the slow rate of decline is still a concern.

The option of restricting and reducing antibiotic use in agriculture may be effective in reducing resistance but is not effective for all antibiotic resistances. Prohibiting subtherapeutic antibiotic use will not immediately reduce AMR in the local bacterial population and will most likely result in reduced production for a period of time; thus, the use of phage therapy can be a viable substitute during this time.

## 3. Phage Infection and Replication

Phage therapy relies on bacteriophages, viruses with specialized structures that enable recognition of specific bacterial species and binding of phages onto the surface of bacteria. The tail structure on phages is responsible for recognition and binding to bacterial surface structures. Notable receptors include O-antigens (O polysaccharide) of lipopolysaccharides (LPS) of gram-negative bacteria, the OmpC protein found on *E. coli* [35] and wall teichoic acids of gram-positive bacteria [36]. This is accomplished by receptor-binding protein (RBP) domains present on tail fibers and is responsible for the high specificity of phages to single families of host bacteria.

Phages of the *Myoviridae* family, which commonly infect *E. coli*, possess a tail structure that contracts after recognition of O-antigens on LPS on the surface of *E. coli*. Irreversible binding, via tail fibers that extend upon recognition of host cells, triggers conformational changes of the base plate, assembly of the sheath and initiation of sheath contraction [35,37]. The contraction punctures the outer cell membrane, while the tip of the “needle” in the sheath contains lysozyme domains that break down the peptidoglycan layer, enabling direct transfer of viral genomic material into the cytoplasm of the bacteria [38]. The *Podoviridae* and *Siphoviridae* families of phages also possess a tail structure used for recognition and binding to O antigens. However, the *Podoviridae* and *Siphoviridae* families do not possess a contractile mechanism; instead, the internal “head” proteins possess lysozyme domains and other viral proteins that extend to create a “tunnel” through the outer membrane and peptidoglycan layer of gram-negative bacteria, enabling the transfer of genetic material into the cytoplasm [39].

A unique trait of phage therapy is the ability to self-replicate during treatment, thereby requiring much smaller and less frequent doses than antibiotics. Phages are parasitic and can only replicate with a host present; thus, phage densities used in phage therapy can vary depending on how it is achieved. Methods involving the application of phages at initial doses with effective densities are considered passive treatments.

Active treatments use the mechanism of bacteriophage replication to attain effective phage densities in situ. While certain situations may require multiple doses, in vivo experiments have demonstrated the ability of single or low doses to exhibit significant bactericidal effects. For example, Salp572-Phage 1 demonstrated exponential replication when the bacterial density threshold was reached, effectively reducing bacterial concentrations and conferring protection against infection and enabling survival [40]. However, it is worth noting that in this study, some of the mice treated with phages alone were unable to clear the entire population of *S. aureus*. The phages worked as prophylactics in that they were able to control and reduce the bacterial population to prevent disease progression and death but required additional treatment or the host’s immune system to finish off the remaining bacteria. With a higher concentration of bacteria, the latent period of phage therapy can be shortened, enabling a higher burst size or plaque-forming units (PFU) per bacterium [41]. Regardless, initial phage density must still surpass a certain minimum threshold due to natural decline in initial phage populations due to several factors, such as degradation or elimination by the organism’s immune system, as well as bacterial densities insufficient to sustain phage replication [42,43]. As there is little evidence of adverse effects from high doses of phage therapy, high concentrations and multiple doses are recommended to result in a higher likelihood of complete clearance of bacterial populations. Since larger initial phage titers and subsequent doses can increase the multiplicity of infection (MOI), there will be a higher probability of establishing a sufficient phage population to avoid the risk of inactivation. For example, in vivo experiments on *S. aureus* in mouse models with an input MOI of 10 and an input MOI of 100 in *Vibrio vulnificus*-infected mice conferred significant protection, but the lower dose had a higher probability of failing to reach the critical threshold needed for self-sustaining replication and was ineffective, leading to the death of the mice [44,45]. This signifies the importance of stricter regulations to prevent personal dosage applications. To avoid repeating the circumstances that led to the widespread AMR in agriculture, prescriptions and courses of treatments should be monitored by specialized personnel, such as veterinarians. In addition to professional observation, a method of applying phages in concentrations rather than MOIs was proposed. This suggestion recommended that sustaining the population and concentration of phages is more vital to bacterial clearance than reaching a specified MOI [46]. To supplement this idea, several factors were suggested to be prioritized during the determination of phage dosages, such as the absolute doses applied and the frequency of doses [46]. These factors are more easily measurable, controllable, and verifiable, thus allowing for better estimation of current phage populations and remaining bacteria [46]. With advances in real-time quantification monitoring technology, such as droplet digital PCR, methods that are rapid, repeatable and have low material costs exist to study factors, such as phage adsorption, time-to-lysis, and phage and bacterial populations in vivo in real-time during the course of treatment [47]. Thus, singular phage doses can be viable with proper monitoring during the course of treatment by trained professionals. With further development and improvements in real-time monitoring technology, phage therapy will have an increased rate of success and be easily implemented with veterinary assistance.

Another complication regarding the practicality of phage therapy is that it requires consideration of the timing for phage application, as different phages exhibit varying periods for optimal activity. For example, phage MSa against *S. aureus* showed the highest activity when administered 10 days after infection, but phage CK-2 required administration within 3 h of bacterial infection for effective bacterial clearance [45]. Mathematical equations and models have been created to understand bacteriophage dynamics and address this complication [48,49]. Equations were devised to calculate the various thresholds critical to successful phage therapy, such as the proliferation threshold for phage replication, clearance threshold for passive therapy, or critical inoculation size for active therapy [49]. Although variation can still be seen among individual cases, these equations highlight the factors that can be improved to increase the efficacy of phage therapy and the importance of bacterial density and optimal timing for the successful clearance of bacteria. Further research is needed to discover more efficient methods to determine phage dosing and the most effective time window for administering different phages.

## 4. Bactericidal Effects of Bacteriophages Demonstrated in Agriculture

Bacteriophages kill bacteria through their lytic replication cycle, where the release of phage progeny results in lysis of the bacterial membrane and subsequent death of the bacterium. Phages have been proven extensively in vitro to exhibit significant bacterial inhibition and bactericidal effects against various disease-causing bacteria within humans and agricultural animals [40,44,45,50,51,52,53]. In vitro studies have demonstrated that combining several phages into cocktails increased bactericidal effectiveness and the range against multiple serotypes of a bacterial species, resulting in increased effectiveness in clearing bacterial populations. A practical application of a bacteriophage cocktail was demonstrated in vitro in which they assessed the therapeutic efficacy of a cocktail of three *E. coli* phages, vB_EcoM_SYGD1, vB_EcoP_SYGE1 and vB_EcoM_SYGMH1, in treating cow mastitis caused by drug-resistant *E. coli* [54]. The phages used in the cocktail were stable at various temperatures, pH values, and chloroform values, demonstrating possible candidates for phage therapy [54]. The benefits of phage cocktails are a reduction in the development of phage resistance, improvement in symptoms, antimicrobial activity and similar effects to antibiotics when applied.

**Figure 2:**
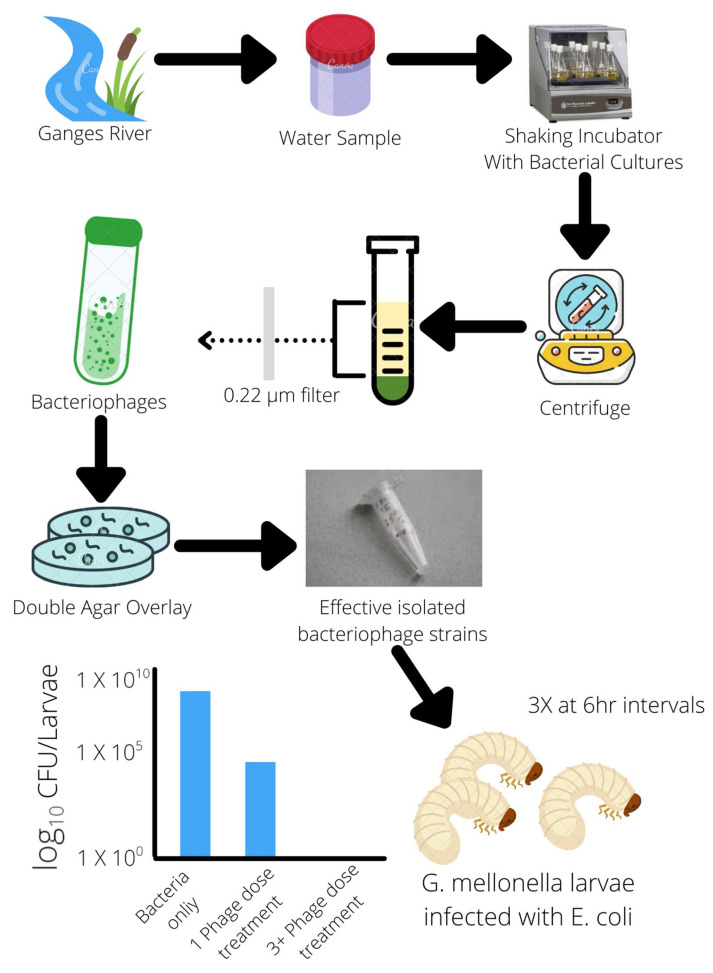
Isolation of bacteriophages from the natural environment effective against *E. coli*.

Multiple in vivo studies of phage therapy using animal models have also been conducted in recent years. These findings highlight not only the specificity of phages to specific bacteria, avoiding unintended target cells but also the stability of phages within different environments and conditions. It is ideal for oral administration in capsules [51,55]. This adaptability and survivability of phages in different conditions may also reflect their natural environments from which most phages are found and isolated; for example, several phage strains were found and isolated from swine-fecal sewage as well as wastewater from poultry slaughterhouses. Their effectiveness against multiple strains of *S. aureus* (18 strains) and *E. coli* (3 strains) was demonstrated in vivo in mice and broiler chickens, clearing infections of MRSA *S. aureus* and *E. coli* colibacillosis [43,51]. Another phage strain isolated from the natural environment of the Ganges River was found to have a broad host range of 31 different *E. coli* strains and serotypes. It was able to demonstrate effective bactericidal effects in reducing *E. coli* populations within an in vivo mouse model. This phage strain exhibited 100% bacterial clearance when three doses were given at 6-h intervals [56] (Figure 2). Effective and specific-targeting phages can be readily isolated from nearby natural environments and cultivated to create doses of phage treatments, and with advances in molecular and gene editing techniques, phage characteristics and virulence can be altered and enhanced more easily than the development of new classes of antibiotics.

As mentioned earlier, several countries have approved the use of several phage products in agriculture and food processing. Recent studies have shown high success in the ability of phages to suppress and control pathogenic bacterial populations within farms and processing facilities. Several phage cocktails have been made against several common species of food-borne bacteria, for example, EcoShieldTM against *E. coli*, Listex P100TM against *Listeria*, and SalmoFREETM against *Salmonella*. These phage cocktails have been studied at various points of the food supply chain, from *Salmonella* in commercial poultry farms to processing plants for ready-to-eat foods. All have demonstrated an effective ability to control bacterial populations in livestock and reduce pathogens in food products to legal standards [57,58,59]. The UK and USA have deemed phages “safe for consumption”, and the USA has even allowed certain phage products against *Listeria*, *Salmonella*, and *E. coli* to be used as food preservatives and be applied directly to food products [60]. However, problems can arise with unregulated use and lack of veterinary surveillance, similar to antibiotics. While predicted to be capable of reducing and clearing bacterial concentrations in contaminated foods, as mentioned in a report by the EFSA Panel on Biological Hazards, with unmonitored usage of phage products and in unspecified doses, the ability of the phage products to reduce bacterial populations in food products can become more varied and unreliable. Without the proper determination of bacterial concentrations by professionals and veterinarians, the applied dosage of the phage products may sometimes be insufficient in clearing bacterial populations and may not be sufficient to prevent recontamination; thus, it can only be currently used as a processing aid [58]. A major benefit is that the possibility of phage resistance and persistence in the environments of processing facilities is considerably low and even minimal when paired with proper disinfection protocols and adequate disposal of unsold treated food products [60,61]. While current phage products may possess certain shortcomings, their ability to reduce pathogenic bacteria in the food supply chain is undisputed. As more data and further research are done to address these issues, phage cocktails can be a promising aid or replacement for antibiotic use in agriculture and the food supply chain.

Plants are known to suffer infections from pests, fungi, and bacteria. Thus, in addition to pesticides and antimicrobials, common antibiotics are used to combat bacterial infections. As with livestock bacterial pathogens, a variety of common antibiotics used in humans are also used to combat plant pathogens, namely, *Pseudomonas* spp. and *Erwinia* spp. Strains from both species were found to possess resistance to high levels of streptomycin (1000 micrograms/mL) [62] and tomato-infecting strains of *Pseudomonas* spp. possess additional resistance to ampicillin and chloramphenicol [63]. With a high rate of AMR in *Pseudomonas* spp. observed in recent years, the risk for blight outbreaks in fruit crops is ever increasing; thus, the usage of phage therapy can serve to mitigate this issue and bypass any current resistance mechanisms employed by *Pseudomonas* spp. In vitro experiments with infected kiwi and tomato plants have shown rapid decreases in bacterial populations and symptoms, including damage to leaf tissues [64,65]. Furthermore, phages have shown additional benefits over antibiotics, as they exhibit a wide tolerance to environmental conditions [64] and selective targeting, avoiding lysis of beneficial Pseudomonas strains [65].

Soft rot Enterobacteriaceae (SRE) infections cause blackleg and soft rot diseases, significantly decreasing crop production and yield. T4-related LIMEstone phages infecting *D. solani* were isolated and showed reduced disease incidence and severity, as well as higher yields in laboratory assays and in field experiments [66]. Their experiment showed that LIMEstone phages were very effective at rapidly infecting all *D. solani* strains [66]. A close relative of LIMEstone1, ΦD5, was tested and remained viable in severe environmental conditions previously unsuitable for phage therapy [67]. Phage treatment of tissue culture and compost with ΦD5 resulted in high levels of protection against infection in potato crops. ΦD5 was shown to have the potential to be used as a biological control measure against *Dickeya* spp. caused soft rot and blackleg [67].

A cocktail of 46 new bacteriophages was created to be used for biocontrol of *D. solani,* and their efficacy in treating soft rot in potatoes under simulated storage conditions was observed [68]. They showed that phage treatment significantly lowered soft rot disease incidence and severity. supporting the use of a phage cocktail in reducing and controlling *D. solani* populations and its spread in potato crops [68]. With further in vivo studies and field trials, phage therapy demonstrates potential in treating bacterial crop diseases. Biochar increases phage adsorption of antibiotic-resistant *E. coli* and *Pseudomonas aeruginosa* bacteria by adsorbing the bacteria in the biochar, increasing the bacterial density and the bactericidal potential of the polyvalent phages [69]. Combined biochar and polyvalent phage treatment reduced residual levels of K-12 and PAO1 and significantly reduced accumulative levels of ARGs in the roots and leaves of lettuce, improving lettuce quality. An added benefit of the combined treatment is that the addition of biochar was associated with an increase in microbial biodiversity in soil and lettuce and diversity of beneficial soil bacteria [69].

Bacteria employ a variety of defense mechanisms against antibiotics, including producing biofilms and evasion by presiding within host cells. Bacteria produce an extracellular matrix that binds multiple bacteria together into a cooperative community and provides structural stability and a layer of defense that confers resistance to recognition from the host’s immune cells and a multitude of antibiotics. This all resulted in the need for a combination of antibiotics and increased dosages. While biofilms may provide some resistance against phage recognition, some phages isolated from natural water sources have coevolved the ability to penetrate biofilms and infect the underlying bacteria. With optimization in vitro, biofilm production can be inhibited or prevented and even completely degraded within a bacterial population [53]. Studies have begun to suggest the use of phages that produce specialized enzymes that degrade biofilms. These phages can be supplemented with minimal doses of antibiotics to remove the remaining extracellular and intracellular populations of multidrug-resistant bacteria [70]. Phages were found to have the ability to clear bacteria that reside within infected cells, as demonstrated by phage vB_SauM_JS25 clearance of intracellular populations of *S. aureus* from within bovine mammary epithelial cells in vivo [71], providing treatments for persistent agricultural epidemics, which would otherwise require large amounts of strong antibiotics to resolve. Furthermore, biofilms may increase susceptibility against phages, as the clustering of similar clonal bacteria and the dynamics of fluids to carry phages to biofilms may result in increased phage interaction due to the increased size, creating a more accessible target for the phages than individual bacteria [72]. The ability of biofilms to allow bacterial populations to adhere to surfaces and resist a certain degree of chemical and mechanical stress is increasingly problematic in food-borne pathogens that can adhere to surfaces within food processing facilities. Phages can effectively reduce biofilm and bacterial populations that adhere to stainless steel surfaces [73], making them ideal as an aid for reducing bacterial contamination in addition to proper disinfection protocols.

Endolysins are bacteriophage-encoded enzymes that hydrolyze the host cell wall through peptidoglycan degradation and allow for the release of bacteriophage progenies. They are vital for the lytic phage life cycle to occur and in recent years show promise as an alternative to antibiotics. This enzyme has been a topic of focus in sectors, such as food, biotechnology, and human medicine with practical applications in biofilm eradication and antimicrobial function [74]. Resistance to endolysins by bacteria may occur through peptidoglycan modifications or bacterial inhibitor proteins; however, the possibility of this development is rare and has not yet been shown in vitro [75]. Endolysins from phages λSA2 and B30 were found to work synergistically against *Streptococci* in vitro and in milk [76]. In whole milk, λSA2 endolysins showed stronger lytic activity than B30 endolysins against all three *Streptococcus* species used in the experiment [76]. However, λSA2 and B30 endolysins do not have synergistic abilities in mastitis treatment, whereas, with individual endolysin treatment, bacterial concentrations of all three *Streptococcal* species were significantly reduced in the mammary gland [76]. Additional research was performed to engineer a unique enzyme through the removal of the middle amidase domain in LysK, termed LysKΔamidase, and showed strong evidence of antimicrobial activity and biofilm eradication that phage lysin can have applied in vitro [77]. They showed that LysKΔamidase had high activity against *S. aureus* and lytic activity against live MRSA strains as well as methicillin-susceptible *S. aureus*. LysKΔamidase is also safer to apply to the animal body than LysK, and it is very effective in eradicating biofilms produced by MRSA [77]. Thus, phage therapy has been demonstrated to be effective against agricultural pathogenic bacteria and, in some cases, exhibits additional qualities to treat infections that not even traditional antibiotic therapy can.

## 5. Phage Resistance

Similar to the selective pressures induced by antibiotic usage, phage therapy can also potentially induce bacteriophage resistance (BPR) in host bacteria. Bacteria can acquire resistance using several mechanisms that target different stages of the phage replication cycle, such as preventing phage adsorption, preventing DNA entry into the bacterium, destroying phage DNA, and a system that results in the death of the infected bacterium [78]. Modification to surface receptors of bacteria can prevent phage adsorption. Mutations within genes responsible for the O-antigens that phages use as receptors can alter the surface layer of the bacterium via glycosylation or acetylation, conferring resistance to specific phages. An experiment showed evidence supporting this possibility; with modifications, such as loss of terminal glucose residues in LPS, T4 phages were unable to adsorb to the bacterial surface [79]. However, these mutations came with a fitness cost, as O antigen truncation in LPS resulted in increased outer membrane permeability to different compounds, especially surfactants, such as SDS [79]. It also caused the bacteria to suffer from a reduced growth rate and longer doubling intervals [79]. When tested in an in vivo model, *Salmonella* strains that gained BPR could not infect the mouse model and were cleared by the host’s immune system [40].

**Figure 3:**
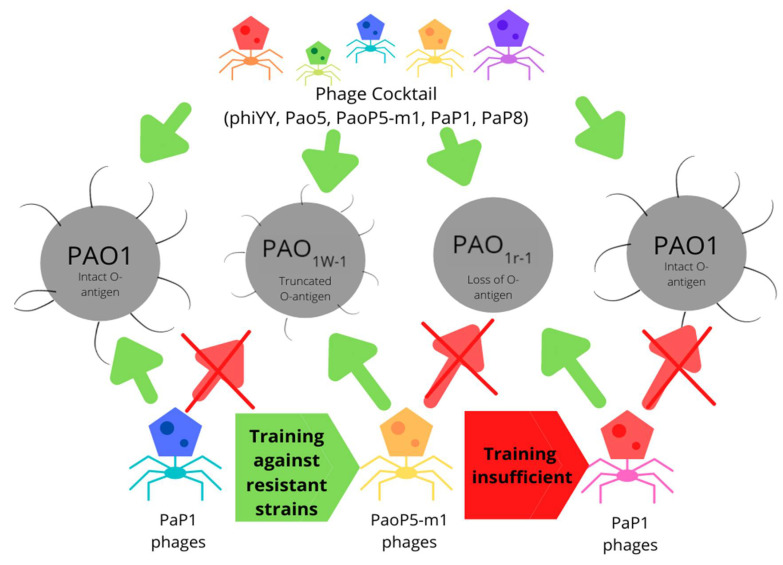
Training of strains effective against phage-resistant bacterium employing truncated O-antigen as a resistance mechanism. Phage cocktails demonstrate effectiveness against all wild-type and resistant strains tested.

Additionally, surface mutations are not able to confer protection against all phage types, a notable example being a strain of *E. coli* 4 sI mutants that gained resistance to G7C phages by reducing the number of O-antigens present and reducing acetylation but suffered from increased susceptibility to T5 phages. Furthermore, several other phage types were found to be effective against wild-type and 4 sI mutants; thus, phage resistance can be easily overcome with the use of phage cocktails [80]. Despite truncation of the O-antigen resistance mechanisms, cultivation of previously ineffective phages with the new resistant bacterium resulted in dozens of strains of mutated phage strains that overcame the resistance and expanded their host range [50] (Figure 3).

There are several limitations associated with these methods for dealing with BPR, including the inability to recultivate a phage strain that can infect in the complete absence of the O-antigen, the regulations of SDS levels in food, inconsistent mutations in membrane permeabilities affecting the practical applications of a phage-SDS combination therapy, and that these effects are only mediated by nonionic surfactants [78,79]. However, some of these limitations can be circumvented with phage cocktails combined with surfactant and their potential use in the disinfection of food processing plants rather than direct application to food products. Future research should investigate the efficacy of currently approved phage products with surfactants approved by food safety agencies.

The revolutionary CRISPR-Cas system was derived from an antibacteriophage system utilized by bacteria. The system is analogous to an adaptive immune system, which detects and destroys phage DNA within the bacterium by first incorporating phage DNA into the CRISPR sequence as spacers located in the bacterial genome and producing RNA that guides the molecular machinery to phage DNA for cleavage and destruction [78,81]. This mechanism is quite effective; however, specific bacteriophages are also coevolving mechanisms to combat the CRISPR-Cas system. For example, phages against *Streptococcus thermophilus* are coevolving and are capable of evading spacer detection using single nucleotide base mutations and/or deletions. By only inducing silent nucleotide changes, there will be no changes to the viral proteins and replicative ability of the virus, but the difference in genetic sequence will allow the viral DNA to evade the CRISPR guide RNA [82]. As mentioned, the CRISPR-Cas system is utilized for genetic editing, and through laboratory modifications to the tail spikes of phages, we have been able to increase the host range. Bactericidal effects of phages against an in vivo mouse model of biofilm-forming *S. aureus* causing soft tissue infection [83] Thus, gene editing techniques are among some of the mechanisms we could employ to modify and create new phage strains to combat antibiotic-resistant bacteria.

Complicating the trade-off of BPR acquisition, mutations to the LPS and antibiotic efflux pump, used by phages for infection, in *E. coli* as BPR mechanisms would result in reduced resistance to certain antibiotics, such as tetracycline. However, some LPS mutations resulted in increased resistance to tetracycline, although at the same time reduced resistance to colistin and overall reduced fitness compared to efflux pump mutants. Similarly, the development of resistance to colistin in *E. coli* resulted in reduced resistance to phages [84,85], indicating high fitness costs of either resistance to antibiotics or phages and the inability to sustain both concurrently, supporting the idea for combination therapy of both phages and antibiotics for highly resistant bacteria.

## 6. Advantages of Phage Therapy over Conventional Antibiotics

One main challenge of phage therapy is how readily available the technology is to be used in practical settings. Phages are a broad classification and often have different optimal functioning conditions and storage condition requirements, such as pH, temperature, and storage media. Several recently discovered and isolated phages, such as pSa-3 and a three *E. coli* phage cocktail comprised of vB_EcoM_SYGD1, vB_EcoP_SYGE1 and vB_EcoM_SYGMH, can survive at high temperatures and pH and stability at various temperatures, pH values and chloroform values, respectively [54]. The broad-spectrum activity of endolysins, such as LysKΔamidase and its broad pH range of 3 to 11 also make it ideal for a variety of applications [77]. Phage banks have recently been established as long-term storage of phages, allowing timely revival for research and application. However, varying tolerances to freezing temperatures and growth media and long-term storage of phages pose a potential problem to both purities of the isolates and may decrease the viability by up to 20% under improper glycerol storage conditions [86].

However, phages do exhibit an advantageous characteristic over antibiotics, which is the ability to work synergistically with other compounds. This was further demonstrated when phage pSa-3 combined with a surfactant, Tween 20, was tested against *S. aureus* aggregates in vitro and in vivo. Tween 20 was found to have prevented *S. aureus* aggregation and increased the adsorption rate and biofilm degradation ability of phage pSa-3 [87]. This suggests that there are accessory compounds that can be administered alongside phage therapy to increase effectiveness, as opposed to antibiotic therapies that increase effectiveness by including more classes of antibiotics.

Recent discoveries found phage’s potential for biopreservation to extend shelf life for food at risk of spoilage due to pathogenic bacteria [88]. The conventional method of biopreservation can be faulty, as they are unreliable in protecting foods from decay caused by bacteria and run the risk of possible pathogens transferring to the consumer, but phage products can be designed to be stable at various temperatures and control bacterial population growth. A study looking at the effects of biopreservation in chilled fish discovered that traditional methods caused higher incidences of alimentary infections and led to the rapid formation of AMR in the fish. When bacteriophage cocktails were used in biopreservation, bacterial degradation was delayed by up to 3 days longer than conventional methods [89]. It was also observed that chilled pork exposed to phage treatment had significantly reduced *Salmonella* populations while also reducing odor and extending the shelf life of the pork up to 14 days [90]. Through rigorous testing, it can be determined that the shelf life can be extended using FDA-approved phages versus temperate phages that could change the bacterial genome without killing them, which would run the risk of further AMR [91,92].

Antibiotics used at subtherapeutic levels improve growth rate and efficiency and improve reproductive performance while also reducing mortality and morbidity [93,94]. Higher intermediate levels have been key to preventing diseases, and even higher therapeutic levels can treat diseases in animals, which is why they are so integral for many feeding programs [93,94]. However, a common theme when talking about antibiotics is antibiotic resistance. Due to the importance of antibiotics to various industries, the decreased effectiveness of antibiotic treatment would be disastrous, as the world already shifts to a “post-antibiotic era” [95,96].

As mentioned previously, bacteriophages are highly selective and will only reduce target bacterial populations, thus ignoring beneficial commensal bacteria residing in the microbiome of livestock. In addition to different bacterial families, the host range of phages can be specified to differentiate between pathogenic and nonpathogenic strains within the same bacterial species [97]. Phage therapy can directly affect pathogens without any side effects to the microbiome, but antibiotics can conversely cause collateral damage as they disrupt the microbiome and surrounding structures [95,96]. This selectivity was further demonstrated in mouse models, in which only foodborne bacteria, such as *E. coli, Salmonella,* and *Listeria* were targeted, while the rest of the microbiome was unaffected [98]. In recent decades, many individuals have been found to develop an allergic response to antibiotics, to which phages can act as an alternative to many who are unable to receive antibiotic therapy [95,96]. This has further safety benefits, as phages are unable to target mammalian cells and thus will not have a direct effect [96]. Phage therapy demonstrates much more targeted treatment than antibiotics, which exhibit indiscriminate elimination of bacteria, including commensal bacteria necessary for the health of many livestock animals.

The increase in new bacteriophage research demonstrates the efficacy of phage cocktails in reducing bacterial populations in the environment and livestock animals. Phage therapy is a good potential alternative to antibiotics sub-therapeutically, since the phage targets a host bacterium and is harmless with virtually no adverse effects or changes to the gut commensal bacteria in animals [43,99,100]. Sub therapeutically, phage therapy is capable of disease prevention, while reversing body weight loss associated with *E. coli* infection [43] and phage cocktails were found to have a synergistic effect with probiotics in improving the average daily feed intake and weight gain of pigs, suggesting a potential for phage therapy to replace antibiotics as growth promoters [101,102,103]. Hence, phages can provide benefits over traditional antibiotics, such as the ability to bypass biofilms and overcome acquired phage resistance, making them great candidates for the agricultural sector and even the medical sector to overcome this crisis of high antibiotic resistance.

## 7. Concluding Remarks

Phage therapy allows bacteriophages, through the lytic cycle, to significantly reduce the number of total antimicrobials required and minimizes antimicrobial resistance genes from being transmitted. Thus, reducing total antimicrobial resistance, especially in common pathogens, such as *E. coli* and Salmonella. In vitro studies show the overall inhibition of bacterial populations against various strains of bacterial species to similar or increased effectiveness to those of antimicrobial drugs. In addition, with the range of conditions and environments, phage therapy is effective. Phage therapy should be considered to enable commercial phage products in food and examine newly developed commercial phage products and their potential. However, more studies should be done to understand its potential in boosting animal productivity and commensal microbiomes in livestock.

## Data Availability

Not applicable.

## References

[1] Wang H., McEntire J.C., Zhang L., Li X., Doyle M. (2012). The transfer of antibiotic resistance from food to humans: Facts, implications and future directions. Rev. Sci. Tech. (Int. Off. Epizoot.).

[2] De Been M., Lanza V.F., de Toro M., Scharringa J., Dohmen W., Du Y., van Schaik W. (2014). Dissemination of cephalosporin resistance genes between *Escherichia coli* strains from farm animals and humans by specific plasmid lineages. PLoS Genet..

[3] Checcucci A., Trevisi P., Luise D., Modesto M., Blasioli S., Braschi I., Mattarelli P. (2020). Exploring the animal waste resistome: The spread of antimicrobial resistance genes through the use of livestock manure. Front. Microbiol..

[4] Antimicrobial Food Processing Aid Uses on Red Meat and Poultry Meat for Which Health Canada has Expressed No Objection. https://www.canada.ca/en/health-canada/corporate/contact-us/publications.html.

[5] Public Health Agency of Canada (2020). Canadian Integrated Program for Antimicrobial Resistance.

[6] Public Health Agency of Canada (2020). Canadian Integrated Program for Antimicrobial Resistance.

[7] World Health Organization (2019). 2019 Antibacterial Agents in Clinical Development: An Analysis of the Antibacterial Clinical Development Pipeline.

[8] Obaidat M.M., Stringer A.P. (2019). Prevalence, molecular characterization, and antimicrobial resistance profiles of Listeria monocytogenes, *Salmonella enterica*, and *Escherichia coli* O157:H7 on dairy cattle farms in Jordan. J. Dairy Sci..

[9] Rasheed M.U., Thajuddin N., Ahamed P., Teklemariam Z., Jamil K. (2014). Antimicrobial drug resistance in strains of *Escherichia coli* isolated from food sources. Rev. Inst. Med. Trop. São Paulo.

[10] Gregova G., Kmet V. (2020). Antibiotic resistance and virulence of *Escherichia coli* strains isolated from animal rendering plant. Sci. Rep..

[11] Varga C., Guerin M.T., Brash M.L., Slavic D., Boerlin P., Susta L. (2019). Antimicrobial resistance in fecal *Escherichia coli* and *Salmonella enterica* isolates: A two-year prospective study of small poultry flocks in Ontario, Canada. BMC Vet. Res..

[12] Gousia P., Economou V., Sakkas H., Leveidiotou S., Papadopoulou C. (2011). Antimicrobial resistance of major foodborne pathogens from major meat products. Foodborne Pathog. Dis..

[13] Oppliger A., Moreillon P., Charrière N., Giddey M., Morisset D., Sakwinska O. (2012). Antimicrobial resistance of *Staphylococcus aureus* strains acquired by pig farmers from pigs. Appl. Environ. Microbiol..

[14] Wang D., Wang Z., Yan Z., Wu J., Ali T., Li J., Lv Y., Han B. (2015). Bovine mastitis *Staphylococcus aureus*: Antibiotic susceptibility profile, resistance genes and molecular typing of methicillin-resistant and methicillin-sensitive strains in China. Infect. Genet. Evol..

[15] Jamali H., Paydar M., Radmehr B., Ismail S., Dadrasnia A. (2015). Prevalence and antimicrobial resistance of *Staphylococcus aureus* isolated from raw milk and dairy products. Food Control.

[16] Li T., Lu H., Wang X., Gao Q., Dai Y., Shang J., Li M. (2017). Molecular characteristics of *Staphylococcus aureus* causing bovine mastitis between 2014 and 2015. Front. Cell. Infect. Microbiol..

[17] MacDougall L., Fyfe M., McIntyre L., Paccagnella A., Cordner K., Kerr A., Aramini J. (2004). Frozen chicken nuggets and strips—A newly identified risk factor for Salmonella Heidelberg infection in British Columbia, Canada. J. Food Prot..

[18] St Amand J.A., Otto S.J., Cassis R., Annett Christianson C.B. (2013). Antimicrobial resistance of Salmonella enterica serovar Heidelberg isolated from poultry in Alberta. Avian Pathol..

[19] Herrera-Sánchez M.P., Rodríguez-Hernández R., Rondón-Barragán I.S. (2020). Molecular characterization of antimicrobial resistance and enterobacterial repetitive intergenic consensus-PCR as a molecular typing tool for *Salmonella* spp. isolated from poultry and humans. Vet. World.

[20] Abd-Elghany S.M., Sallam K.I., Abd-Elkhalek A., Tamura T. (2015). Occurrence, genetic characterization and antimicrobial resistance of *Salmonella* isolated from chicken meat and giblets. Epidemiol. Infect..

[21] Liljebjelke K.A., Hofacre C.L., White D.G., Ayers S., Lee M.D., Maurer J.J. (2017). Diversity of antimicrobial resistance phenotypes in *Salmonella* isolated from commercial poultry farms. Front. Vet. Sci..

[22] Bronowski C., James C.E., Winstanley C. (2014). Role of environmental survival in transmission of *Campylobacter jejuni*. FEMS Microbiol. Lett..

[23] Elhadidy M., Miller W.G., Arguello H., Álvarez-Ordóñez A., Duarte A., Dierick K., Botteldoorn N. (2018). Genetic basis and clonal population structure of antibiotic resistance in *Campylobacter jejuni* isolated from broiler carcasses in Belgium. Front. Microbiol..

[24] Abay S., Kayman T., Otlu B., Hizlisoy H., Aydin F., Ertas N. (2014). Genetic diversity and antibiotic resistance profiles of *Campylobacter jejuni* isolates from poultry and humans in Turkey. Int. J. Food Microbiol..

[25] Khan J.A., Rathore R.S., Abulreesh H.H., Qais F.A., Ahmad I. (2018). Prevalence and antibiotic resistance profiles of *Campylobacter jejuni* isolated from poultry meat and related samples at retail shops in Northern India. Foodborne Pathog. Dis..

[26] Agunos A., Léger D., Avery B.P., Parmley E.J., Deckert A., Carson C.A., Dutil L. (2013). Ciprofloxacin-resistant *Campylobacter* spp. in retail chicken, western Canada. Emerg. Infect. Dis..

[27] United States Food and Drug Administration (2008). Retail Meat Report: National Antimicrobial Resistance Monitoring System. http://www.fda.gov/downloads/AnimalVeterinary/SafetyHealth/AntimicrobialResistance/NationalAntimicrobialResistanceMonitoringSystem/UCM237111.pdf.

[28] Ghosh S., LaPara T.M. (2007). The effects of subtherapeutic antibiotic use in farm animals on the proliferation and persistence of antibiotic resistance among soil bacteria. ISME J..

[29] Kagambèga A., Lienemann T., Aulu L., Traoré A.S., Barro N., Siitonen A., Haukka K. (2013). Prevalence and characterization of *Salmonella enterica* from the feces of cattle, poultry, swine and hedgehogs in Burkina Faso and their comparison to human *Salmonella* isolates. BMC Microbiol..

[30] Montero I., Herrero A., Mendoza M.C., Rodicio R., Rodicio M.R. (2012). Virulence-resistance plasmids (pUO-StVR2-like) in meat isolates of *Salmonella enterica* serovar Typhimurium. Food Res. Int..

[31] Yoshizawa N., Usui M., Fukuda A., Asai T., Higuchi H., Okamoto E., Seki K., Takada H., Tamura Y. (2020). Manure Compost Is a Potential Source of Tetracycline-Resistant *Escherichia coli* and Tetracycline Resistance Genes in Japanese Farms. Antibiotics.

[32] Usui M., Kawakura M., Yoshizawa N., San L.L., Nakajima C., Suzuki Y., Tamura Y. (2017). Survival and prevalence of *Clostridium difficile* in manure compost derived from pigs. Anaerobe.

[33] Zhang Y.J., Hu H.W., Chen Q.L., Singh B.K., Yan H., Chen D., He J.Z. (2019). Transfer of antibiotic resistance from manure-amended soils to vegetable microbiomes. Environ. Int..

[34] Hesp A., Veldman K., van der Goot J., Mevius D., van Schaik G. (2019). Monitoring antimicrobial resistance trends in commensal *Escherichia coli* from livestock, the Netherlands, 1998 to 2016. Eurosurveillance.

[35] Figura G., Budynek P., Dabrowska K. (2010). Bacteriophage T4: Molecular aspects of bacterial cell infection and the role of capsid proteins. Postep. Hig. Med. Dosw..

[36] Baptista C., Santos M.A., São-José C. (2008). Phage SPP1 reversible adsorption to Bacillus subtilis cell wall teichoic acids accelerates virus recognition of membrane receptor YueB. J. Bacteriol..

[37] Taylor N.M., Prokhorov N.S., Guerrero-Ferreira R.C., Shneider M.M., Browning C., Goldie K.N., Stahlberg H., Leiman P.G. (2016). Structure of the T4 baseplate and its function in triggering sheath contraction. Nature.

[38] Kanamaru S., Leiman P.G., Kostyuchenko V.A., Chipman P.R., Mesyanzhinov V.V., Arisaka F., Rossmann M.G. (2002). Structure of the cell-puncturing device of bacteriophage T4. Nature.

[39] Wang C., Tu J., Liu J., Molineux I.J. (2019). Structural dynamics of bacteriophage P22 infection initiation revealed by cryo-electron tomography. Nat. Microbiol..

[40] Capparelli R., Nocerino N., Iannaccone M., Ercolini D., Parlato M., Chiara M., Iannelli D. (2010). Bacteriophage therapy of *Salmonella enterica*: A fresh appraisal of bacteriophage therapy. J. Infect. Dis..

[41] Nabergoj D., Modic P., Podgornik A. (2018). Effect of bacterial growth rate on bacteriophage population growth rate. MicrobiologyOpen.

[42] Abedon S. (2011). Phage therapy pharmacology: Calculating phage dosing. Adv. Appl. Microbiol..

[43] Naghizadeh M., Torshizi MA K., Rahimi S., Engberg R.M., Dalgaard T.S. (2019). Effect of serum anti-phage activity on colibacillosis control by repeated phage therapy in broilers. Vet. Microbiol..

[44] Cerveny K.E., DePaola A., Duckworth D.H., Gulig P.A. (2002). Phage therapy of local and systemic disease caused by *Vibrio vulnificus* in iron-dextran-treated mice. Infect. Immun..

[45] Capparelli R., Parlato M., Borriello G., Salvatore P., Iannelli D. (2007). Experimental phage therapy against *Staphylococcus aureus* in mice. Antimicrob. Agents Chemother..

[46] Abedon S.T. (2016). Phage therapy dosing: The problem(s) with multiplicity of infection (MOI). Bacteriophage.

[47] Morella N.M., Yang S.C., Hernandez C.A., Koskella B. (2018). Rapid quantification of bacteriophages and their bacterial hosts in vitro and in vivo using droplet digital PCR. J. Virol. Methods.

[48] Kasman L.M., Kasman A., Westwater C., Dolan J., Schmidt M.G., Norris J.S. (2002). Overcoming the phage replication threshold: A mathematical model with implications for phage therapy. J. Virol..

[49] Payne R.J., Jansen V.A. (2001). Understanding bacteriophage therapy as a density-dependent kinetic process. J. Theor. Biol..

[50] Yang Y., Shen W., Zhong Q., Chen Q., He X., Baker J.L., Xiong K., Jin X., Wang J., Hu F. (2020). Development of a bacteriophage cocktail to constrain the emergence of phage-resistant Pseudomonas aeruginosa. Front. Microbiol..

[51] Wang Z., Zheng P., Ji W., Fu Q., Wang H., Yan Y., Sun J. (2016). SLPW: A virulent bacteriophage targeting methicillin-resistant *Staphylococcus aureus* in vitro and in vivo. Front. Microbiol..

[52] Porter J., Anderson J., Carter L., Donjacour E., Paros M. (2016). In vitro evaluation of a novel bacteriophage cocktail as a preventative for bovine coliform mastitis. J. Dairy Sci..

[53] Yulinery T., Triana E., Suharna N., Nurhidayat N. (2019). Isolation and anti-*Escherichia coli* biofilm activity of lytic bacteriophages isolated from water environment in vitro. IOP Conference Series: Earth and Environmental Science.

[54] Guo M., Gao Y., Xue Y., Liu Y., Zeng X., Cheng Y., Ma J., Wang H., Sun J., Wang Z. (2021). Bacteriophage Cocktails Protect Dairy Cows against Mastitis Caused By Drug Resistant *Escherichia coli* Infection. Front. Cell. Infect. Microbiol..

[55] Ramirez K., Cazarez-Montoya C., Lopez-Moreno H.S., Castro-del Campo N. (2018). Bacteriophage cocktail for biocontrol of *Escherichia coli* O157:H7: Stability and potential allergenicity study. PLoS ONE.

[56] Manohar P., Tamhankar A.J., Lundborg C.S., Ramesh N. (2018). Isolation, characterization and in vivo efficacy of Escherichia phage myPSH1131. PLoS ONE.

[57] Clavijo V., Baquero D., Hernandez S., Farfan J.C., Arias J., Arévalo A., Donado-Godoy P., Vives-Flores M. (2019). Phage cocktail SalmoFREE^®^ reduces Salmonella on a commercial broiler farm. Poult. Sci..

[58] Carter C.D., Parks A., Abuladze T., Li M., Woolston J., Magnone J., Senecal A., Kropinski A.M., Sulakvelidze A. (2012). Bacteriophage cocktail significantly reduces *Escherichia coli* O157:H7 contamination of lettuce and beef, but does not protect against recontamination. Bacteriophage.

[59] Miguéis S., Saraiva C., Esteves A. (2017). Efficacy of LISTEX P100 at different concentrations for reduction of *Listeria monocytogenes* inoculated in sashimi. J. Food Prot..

[60] Fernández L., Gutiérrez D., Rodríguez A., García P. (2018). Application of bacteriophages in the agro-food sector: A long way toward approval. Front. Cell. Infect. Microbiol..

[61] EFSA Panel on Biological Hazards (BIOHAZ) (2016). Evaluation of the safety and efficacy of Listex™ P100 for reduction of pathogens on different ready-to-eat (RTE) food products. EFSA J..

[62] Laforest M., Bisaillon K., Ciotola M., Cadieux M., Hébert P.O., Toussaint V., Svircev A.M. (2019). Rapid identification of Erwinia amylovora and *Pseudomonas syringae* species and characterization of E. amylovora streptomycin resistance using quantitative PCR assays. Can. J. Microbiol..

[63] Hwang M.S., Morgan R.L., Sarkar S.F., Wang P.W., Guttman D.S. (2005). Phylogenetic characterization of virulence and resistance phenotypes of Pseudomonas syringae. Appl. Environ. Microbiol..

[64] Flores O., Retamales J., Núñez M., León M., Salinas P., Besoain X., Yañez C., Bastías R. (2020). Characterization of bacteriophages against *Pseudomonas syringae* pv. actinidiae with potential use as natural antimicrobials in kiwifruit plants. Microorganisms.

[65] Rabiey M., Roy S.R., Holtappels D., Franceschetti L., Quilty B.J., Creeth R., Sundin G.W., Wagemans J., Lavigne R., Jackson R.W. (2020). Phage biocontrol to combat *Pseudomonas syringae* pathogens causing disease in cherry. Microb. Biotechnol..

[66] Adriaenssens E.M., van Vaerenbergh J., Vandenheuvel D., Dunon V., Ceyssens P.J., de Proft M., Kropinski A.M., Noben J.P., Maes M., Lavigne R. (2012). T4-Related Bacteriophage LIMEstone Isolates for the Control of Soft Rot on Potato Caused by ‘Dickeya solani’. PLoS ONE.

[67] Czajkowski R., Smolarska A., Ozymko Z. (2017). The viability of lytic bacteriophage ΦD5 in potato-associated environments and its effect on *Dickeya solani* in potato (*Solanum tuberosum* L.) plants. PLoS ONE.

[68] Carstens A.B., Djurhuus A.M., Kot W., Jacobs-Sera D., Hatfull G.F., Hansen L.H. (2018). Unlocking the Potential of 46 New Bacteriophages for Biocontrol of *Dickeya solani*. Viruses.

[69] Ye M., Sun M., Zhao Y., Jiao W., Xia B., Liu M., Feng Y., Zhang Z., Huang D., Huang R. (2018). Targeted inactivation of antibiotic-resistant *Escherichia coli* and *Pseudomonas aeruginosa* in a soil-lettuce system by combined polyvalent bacteriophage and biochar treatment. Environ. Pollut..

[70] Kolenda C., Josse J., Medina M., Fevre C., Lustig S., Ferry T., Laurent F. (2020). Evaluation of the activity of a combination of three bacteriophages alone or in association with antibiotics on *Staphylococcus aureus* embedded in biofilm or internalized in osteoblasts. Antimicrob. Agents Chemother..

[71] Zhang L., Sun L., Wei R., Gao Q., He T., Xu C., Liu X., Wang R. (2017). Intracellular *Staphylococcus aureus* control by virulent bacteriophages within MAC-T bovine mammary epithelial cells. Antimicrob. Agents Chemother..

[72] Abedon S.T. (2012). Spatial vulnerability: Bacterial arrangements, microcolonies, and biofilms as responses to low rather than high phage densities. Viruses.

[73] Islam M., Zhou Y., Liang L., Nime I., Liu K., Yan T., Wang X., Li J. (2019). Application of a phage cocktail for control of Salmonella in foods and reducing biofilms. Viruses.

[74] Abdelrahman F., Easwaran M., Daramola O.I., Ragab S., Lynch S., Oduselu T.J., Khan F.M., Ayobami A., Adnan F., Torrents E. (2021). Phage-Encoded Endolysins. Antibiotics.

[75] Grishin A.V., Karyagina A.S., Vasina D.V., Vasina I.V., Gushchin V.A., Lunin V.G. (2020). Resistance to peptidoglycan-degrading enzymes. Crit. Rev. Microbiol..

[76] Schmelcher M., Powell A.M., Camp M.J., Pohl C.S., Donovan D.M. (2015). Synergistic streptococcal phage λSA2 and B30 endolysins kill streptococci in cow milk and in a mouse model of mastitis. Appl. Microbiol. Biotechnol..

[77] Zhou Y., Zhang H., Bao H., Wang X., Wang R. (2017). The lytic activity of recombinant phage lysin LysKΔamidase against staphylococcal strains associated with bovine and human infections in the Jiangsu province of China. Res. Vet. Sci..

[78] Labrie S.J., Samson J.E., Moineau S. (2010). Bacteriophage resistance mechanisms. Nat. Rev. Microbiol..

[79] Zhong Z., Emond-Rheault J.-G., Bhandare S., Lévesque R., Goodridge L. (2020). Bacteriophage-Induced Lipopolysaccharide Mutations in *Escherichia coli* Lead to Hypersensitivity to Food Grade Surfactant Sodium Dodecyl Sulfate. Antibiotics.

[80] Knirel Y.A., Prokhorov N.S., Shashkov A.S., Ovchinnikova O.G., Zdorovenko E.L., Liu B., Letarov A.V. (2015). Variations in O-antigen biosynthesis and O-acetylation associated with altered phage sensitivity in *Escherichia coli* 4 s. J. Bacteriol..

[81] Szczepankowska A. (2012). Role of CRISPR/cas system in the development of bacteriophage resistance. Adv. Virus Res..

[82] Deveau H., Barrangou R., Garneau J.E., Labonté J., Fremaux C., Boyaval P., Romero D.A., Horvath P., Moineau S. (2008). Phage response to CRISPR-encoded resistance in *Streptococcus thermophilus*. J. Bacteriol..

[83] Cobb L.H., Park J., Swanson E.A., Beard M.C., McCabe E.M., Rourke A.S., Seo K.S., Olivier A.K., Priddy L.B. (2019). CRISPR–Cas9 modified bacteriophage for treatment of *Staphylococcus aureus* induced osteomyelitis and soft tissue infection. PLoS ONE.

[84] Burmeister A.R., Fortier A., Roush C., Lessing A.J., Bender R.G., Barahman R., Grant R., Chan B.K., Turner P.E. (2020). Pleiotropy complicates a trade-off between phage resistance and antibiotic resistance. Proc. Natl. Acad. Sci. USA.

[85] Hao G., Chen A.I., Liu M., Zhou H., Egan M., Yang X., Kan B., Wang H., Goulian M., Zhu J. (2019). Colistin resistance-mediated bacterial surface modification sensitizes phage infection. Antimicrob. Agents Chemother..

[86] Anand T., Virmani N., Bera B.C., Vaid R.K., Kumar A., Tripathi B.N. (2020). Applications of Personalized Phage Therapy highlighting the importance of Bacteriophage Banks against Emerging Antimicrobial Resistance. Def. Life Sci. J..

[87] Kim S.G., Giri S.S., Yun S., Kim H.J., Kim S.W., Kang J.W., Han S.J., Kwon J., Oh W.T., Jun J.W. (2020). Synergistic phage–surfactant combination clears IgE-promoted *Staphylococcus aureus* aggregation in vitro and enhances the effect in vivo. Int. J. Antimicrob. Agents.

[88] Połaska M., Sokołowska B. (2019). Review bacteriophages—A new hope or a huge problem in the food industry. AIMS Microbiol..

[89] Zulkarneev E.R., Aleshkin A.V., Kiseleva I.A., Rubalsky E.O., Rubalsky O.V. (2019). Bacteriophage Cocktail Effectively Prolonging the Shelf-Life of Chilled Fish. Bull. Exp. Biol. Med..

[90] Wang C., Yang J., Zhu X., Lu Y., Xue Y., Lu Z. (2017). Effects of Salmonella bacteriophage, nisin and potassium sorbate and their combination on safety and shelf life of fresh chilled pork. Food Control.

[91] Kahn L.H., Bergeron G., Bourassa M.W., De Vegt B., Gill J., Gomes F., Malouin F., Opengart K., Ritter G.D., Singer R.S. (2019). From farm management to bacteriophage therapy: Strategies to reduce antibiotic use in animal agriculture. Ann. N. Y. Acad. Sci..

[92] Adesanya O., Oduselu T., Akin-Ajani O., Adewumi O.M., Ademowo O.G. (2020). An exegesis of bacteriophage therapy: An emerging player in the fight against antimicrobial resistance. AIMS Microbiol..

[93] Cromwell G.L. (2002). Why and how antibiotics are used in swine production. Anim. Biotechnol..

[94] Hays V.W., Moats W.A. (1986). Benefits and risks of antibiotics use in agriculture. Agricultural Uses of Antibiotics.

[95] Gordillo Altamirano F.L., Barr J.J. (2019). Phage therapy in the postantibiotic era. Clin. Microbiol. Rev..

[96] Abedon S.T., García P., Mullany P., Aminov R. (2017). Editorial: Phage therapy: Past, present and future. Front. Microbiol..

[97] Tomat D.D., Migliore L., Aquili V., Quiberoni A.D.L., Balagué C. (2013). Phage biocontrol of enteropathogenic and shiga toxin-producing *Escherichia coli* in meat products. Front. Cell. Infect. Microbiol..

[98] Dissanayake U., Ukhanova M., Moye Z.D., Sulakvelidze A., Mai V. (2019). Bacteriophages reduce pathogenic *Escherichia coli* counts in mice without distorting gut microbiota. Front. Microbiol..

[99] Cani P.D., Possemiers S., Van de Wiele T., Guiot Y., Everard A., Rottier O., Geurts L., Naslain D., Neyrinck A., Lambert D.M. (2009). Changes in gut microbiota control inflammation in obese mice through a mechanism involving GLP-2-driven improvement of gut permeability. Gut.

[100] Cieplak T., Soffer N., Sulakvelidze A., Nielsen D.S. (2018). A bacteriophage cocktail targeting *Escherichia coli* reduces *E. coli* in simulated gut conditions 2018, while preserving a nontargeted representative commensal normal microbiota. Gut Microbes.

[101] Kim K.H., Ingale S.L., Kim J.S., Lee S.H., Lee J.H., Kwon I.K., Chae B.J. (2014). Bacteriophage and probiotics both enhance the performance of growing pigs but bacteriophage are more effective. Anim. Feed. Sci. Technol..

[102] Kim J.S., Hosseindoust A., Lee S.H., Choi Y.H., Kim M.J., Lee J.H., Kwon I.K., Chae B.J. (2017). Bacteriophage cocktail and multistrain probiotics in the feed for weanling pigs: Effects on intestine morphology and targeted intestinal coliforms and Clostridium. Animal.

[103] Hosseindoust A.R., Lee S.H., Kim J.S., Choi Y.H., Noh H.S., Lee J.H., Jha P.K., Kwon I.K., Chae B.J. (2017). Dietary bacteriophages as an alternative for zinc oxide or organic acids to control diarrhea and improve the performance of weanling piglets. Vet. Med..

